# A Prior History of Substance Abuse in Veterans Undergoing Bariatric Surgery

**DOI:** 10.1155/2013/740312

**Published:** 2013-06-12

**Authors:** Maureen Tedesco, William Q. Hua, Jessica A. Lohnberg, Nina Bellatorre, Dan Eisenberg

**Affiliations:** ^1^Department of Surgery, Stanford School of Medicine, Stanford, CA 94304, USA; ^2^Department of Surgery, Palo Alto VA Health Care System, Palo Alto, CA 94304, USA; ^3^Department of Behavioral Medicine, Palo Alto VA Health Care System, Palo Alto, CA 94304, USA

## Abstract

*Background*. The rates of obesity and substance abuse are high among US veterans. *Objective*. To examine weight loss and substance abuse rates following bariatric surgery in veterans with a history of substance abuse (SA). *Methods*. A prospective database of consecutive bariatric operations was reviewed. Data for SA patients were compared to patients without a substance abuse history (NA). Behavioral medicine staff followed patients throughout the pre- and postoperative courses. *Results*. Of 205 bariatric surgery patients, there were 74 (36.1%) SA patients. The mean preoperative body mass index (BMI) was 46.2 ± 8.1 kg/m^2^, and percent excess weight loss at 12 months was 71.8%, 58.0%, and 33.5% for Roux-en-Y gastric bypass, laparoscopic sleeve gastrectomy, and laparoscopic gastric banding, respectively, not significantly different than the NA group (*P* = 0.15, 0.75, 0.96). Postoperative substance abuse in SA and NA patients was 8.1% and 1.5%, respectively (*P* = 0.234). *Conclusion*. A prior history of substance abuse is common in veterans undergoing bariatric surgery; weight loss results are comparable to the general veteran bariatric cohort. Rates of substance abuse are low postoperatively, but higher in patients without a prior history of substance abuse. Close multidisciplinary followup throughout the postoperative course is likely to be integral to the patient's success.

## 1. Introduction

The prevalence of overweight and obesity is high among veterans and more common in veterans than in the general (nonveteran) population. Nearly 3/4 of the veteran population is at least overweight, and more than 1/3 of this population is obese or morbidly obese [1, 2]. Obese veterans often carry multiple medical and psychiatric comorbid conditions, making it a higher-risk population for weight loss surgery compared to the general population. Nonetheless, bariatric surgery has been shown to be safe and effective in designated veteran medical centers [3, 4]. This population also has a high prevalence of having a history of prior substance abuse. A recent survey of American veterans found the rate of alcohol abuse to be 4.0% and the rate of illicit drug use to be 1.9% [5]. These values include both the current use and/or a recorded diagnosis of substance abuse.

In this study we assessed the prevalence of a history of illicit drug and/or alcohol abuse in the population undergoing bariatric surgery and examined whether a history of substance abuse (SA) impacts weight loss outcomes. We also evaluated illicit drug and alcohol abuse rates in the first 12 months of the postoperative period. The findings in this study are significant as bariatric surgery continues to be a scarce, yet important, treatment modality for the morbidly obese veteran population, with few VA bariatric centers serving the United States. Ongoing substance abuse is a known contraindication to weight loss surgery, but it is yet unclear whether a past history of drug or alcohol abuse impacts bariatric surgery outcomes. We sought to investigate whether patients with a prior history of substance abuse suffer poorer weight loss outcomes from bariatric surgery and whether these patients are at a greater risk for substance abuse following the surgery. This may assist in guiding preoperative screening and postoperative followup for weight loss surgery.

## 2. Methods

After obtaining the Institutional Review Board approval, we retrospectively reviewed a prospectively collected database of patients who had undergone bariatric surgery at the Palo Alto Veterans Affairs (VA) hospital. Patient's demographics including age, gender, body mass index (BMI), and history of alcohol or drug abuse were collected. 

A multidisciplinary team, including bariatricians, psychologists, dieticians, physical therapists, and surgeons, evaluated each patient preoperatively. Patients who had a history of illicit drug abuse were required to participate in a specialized mental health clinic and undergo regular toxicology screening. This included periodic mental health screening by a psychologist and required toxicology testing to ensure sobriety prior to surgery and in the postoperative period as well. A history of drug or alcohol abuse (SA cohort) was defined as any prior inpatient or outpatient treatment for substance abuse, a Diagnostic and Statistical Manual of Mental Disorders-IV text revision diagnosis of substance abuse, or a screening-positive history of abuse that was documented in the medical record. There are several mechanisms in the VA health care system by which patients are screened for current or past history of substance abuse. These include required screening during primary care visits when a standard screening assessment is automatically administered, as well as during a mental health clinic intake, or through any specialty clinic in the VA that has assumed screening as part of their regular practice in addition to that of the primary care. As appropriate, based on the Palo Alto VA screening assessment, a formal diagnosis of substance abuse is subsequently made by a mental health professional after necessary follow-up measures are taken. Once this is complete, a notation is made in the computerized medical record system. The cohort of bariatric surgery patients with no history of drug or alcohol abuse, and negative institutional screening, was designated by “nonsubstance abuse” (NA cohort).

Postoperative followup occurred at regular intervals, at 2 weeks, 2 months, 6 months, 12 months, and annually thereafter. At each postoperative visit, irrespective of SA history, all patients met with each member of the multidisciplinary team, including psychologists of the Behavioral Medicine faculty, at which time they were rescreened for substance abuse. Weights, vital signs, and routine labs were obtained at each visit. A student's *t* test was used to determine differences between means of each group and Fisher's exact test for categorical values. A *P*-value <0.05 was considered statistically significant.

## 3. Results 

Of 205 patients who underwent bariatric surgery at the Palo Alto VA between 2002 and 2011, 74 patients (36.1%) had a history of substance abuse (SA cohort), while 131 patients (63.9%) had no prior history of substance abuse (NA cohort). In the SA group there were 44 former alcohol abusers and 48 former illicit drug abusers (eighteen patients had both a history of alcohol abuse and illicit drug abuse). The average BMI for all patients was 46.2 kg/m^2^ (Table 1), with an average excess body weight of approximately 144.6 lbs (65.7 kg). Average age and gender distribution is shown.

**Figure 1:**
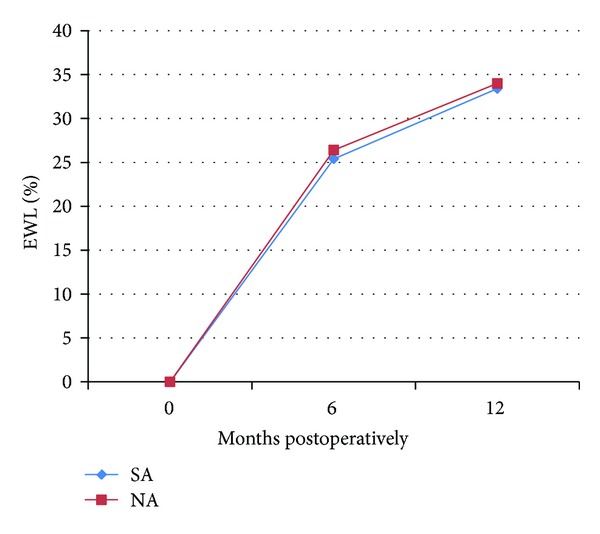
Percent excess weight loss (%EWL) in each group, after gastric banding (LGB). SA: substance abuse cohort; NA: no substance abuse cohort.

 Laparoscopic gastric bands (LGB) accounted for 11.2% of the total operations (23 patients). Of the patients who received LGB, 47.8% had a history of SA (11 patients). Percent excess weight loss (%EWL) at 6 months was 25.4 ± 20.7% in the SA group, compared to 26.4 ± 12.5% for the nonabusers group (NA) (Table 2). At 1 year, %EWL was similar between the SA and NA groups, 33.4 ± 25.2% in the SA group, compared to 34.0 ± 27.2% in the NA group (Figure 1). These differences were not statistically significant.

**Figure 2:**
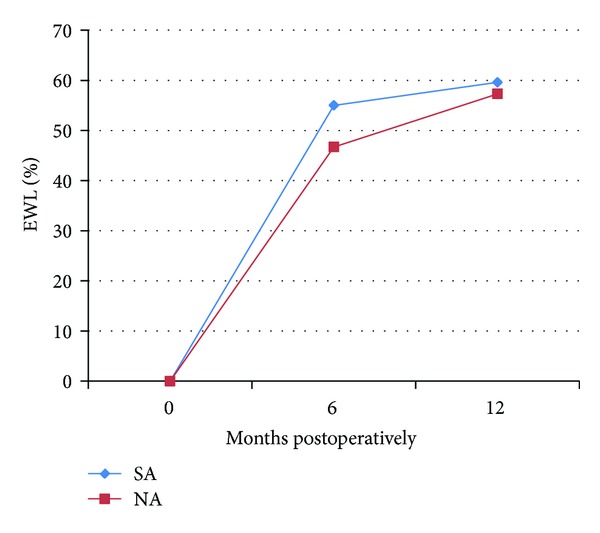
Percent excess weight loss (%EWL) in each group, after sleeve gastrectomy (LSG). SA: substance abuse cohort; NA: no substance abuse cohort.

 Laparoscopic sleeve gastrectomy (LSG) comprised 35% of the operations (total 72). In this group, 30.6% had a history of SA. The SA group trended towards slightly more weight loss than the NA group (Table 2). At 6 months, SA group had a 55.0 ± 25.7%  %EWL, compared to 46.7 ± 17.5% in the NA group (*P* = 0.18). This effect held true at a 1-year followup, although this difference was not significant; the %EWL in the SA group was 59.6 ± 25.2%, compared to 57.3 ± 21.8% in the NA group (Figure 2).

**Figure 3:**
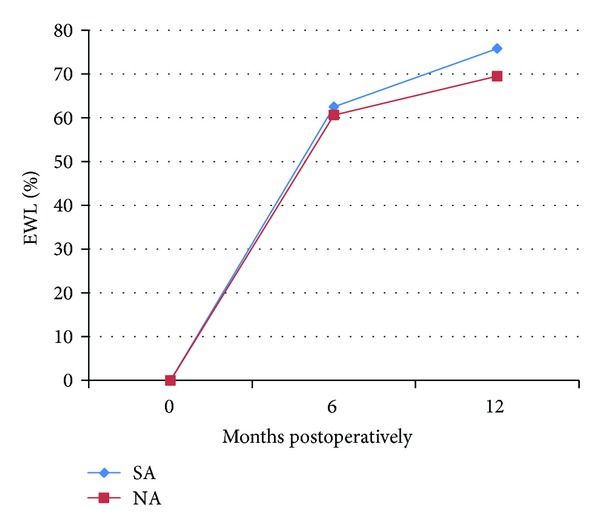
Percent excess weight loss (%EWL) in each group, after Roux-en-Y gastric bypass (RYGB). SA: substance abuse cohort; NA: no substance abuse cohort.

 The remainder of the patients underwent Roux-en-Y gastric bypass (RYGB) (53.7% of total patients). In this group, 37.3% (41 patients) had a history of SA. The SA group demonstrated greater weight loss than the NA group at both 6 and 12 months that was not statistically significant (Table 2). The SA group %EWL was 62.5 ± 19.4% at 6 months and 75.8 ± 23.5% at 12 months, compared to 60.6 ± 23.6% at 6 months and 69.5 ± 19.9% at 1 year in the NA group (*P* = 0.15) (Figure 3).

 Overall, 66% of the SA patients were diabetic at the time of surgery, of which 92% had improvement or remission and 56% had complete remission at 1 year after the surgery. Similarly, 85% had hypertension, with half of the patients being able to discontinue antihypertensive medication 12 months postoperatively. All patients were taking multiple medications preoperatively and had a decrease in total medications prescribed after the surgery.

Eight patients developed substance abuse postoperatively (Table 3); 6 patients abused alcohol, and two patients were using methamphetamines. Six of the eight patients had a prior history of substance abuse, while two patients who had no history of abuse, developed substance abuse postoperatively. To our knowledge, no one required hospitalization for intoxication.

Select markers of nutritional status in this group were in the normal range before the surgery and remained within the normal range 1 year postoperatively in the SA cohort. The mean pre- and postoperative albumin levels were 3.8 and 3.9 g/dL, respectively (*P* = 0.159) (normal range 3.4–5.4 g/dL); the mean pre- and postoperative vitamin B12 levels were 472.9 and 532.2 pg/mL, respectively (*P* = 0.276) (normal range 200–900 pg/mL); the mean pre- and postoperative ferritin levels were 152.9 and 119.0 ng/mL, respectively (*P* = 0.016) (normal range 30–400 ng/mL). Although the decrease of ferritin levels in the postoperative period was statistically significant compared to the preoperative cohort, the levels remained well within the normal reference range and were not clinically significant. No significant difference in the levels of nutritional markers was seen between the SA and NA groups.

## 4. Discussion

Ongoing substance abuse, poorly-controlled depression, or other major psychiatric illness are considered to be a contraindication to bariatric surgery [6]. Although mental health screening is recommended—and common—prior to weight loss surgery, there is no consensus on to the type or structure of psychological assessment. Moreover, generally, the correlation between mental health and postoperative outcomes has not been elucidated. More specifically, there is little data examining the relationship between a prior history of substance abuse and bariatric surgery outcomes. In this population, a multidisciplinary approach may be the key to ensure good surgical outcomes. In this study we have shown that patients with SA exhibit equivalent weight loss at 6 and 12 months compared to the patients who have no history of alcohol and/or drug abuse, which may be a reflection of intensive workup and close followup by a dedicated multidisciplinary team. This study also demonstrates low postoperative substance abuse rates in SA patients. More patients with a history of SA developed illicit drug or alcohol abuse after bariatric surgery compared to their NA counterparts, although we did not identify a statistically significant difference. This, however, may reflect a small number of study subjects.

 American veterans have been found to have a rate of current alcohol abuse of 4.0% and a rate of illicit drug use of 1.9% [5]. These values were derived from a self-reported study, including both current use and/or a previously recorded diagnosis of substance abuse, which may have underestimated actual rates of substance abuse. Our study population, which focused on veterans who had undergone bariatric surgery, had a prevalence of drug and alcohol abuse that is ten times higher than that reported by Chwastiak et al. This may reflect that the data in our study are not simply self-reported but rather relied on specific institutional screening methods, in addition to the electronic medical record. Interestingly, multiple studies report a decreased lifetime incidence of substance abuse among obese patients [7], while others have found no association between BMI and substance abuse [8]. Although we did not specifically compare the prevalence of substance abuse in the morbidly obese cohort at Palo Alto VA and that of a normal weight cohort, we certainly found high rates of prior SA in the bariatric surgical population. Other studies found that obese persons had significantly increased risk of alcohol use disorder, in addition to other psychiatric disorders [8]. 

 This study emphasizes the importance of a thorough mental health screening for bariatric surgery that provides an opportunity for timely intervention and appropriate, individualized postoperative followup. Other psychiatric conditions are also found with higher prevalence in the obese population seeking bariatric surgery. Axis I psychiatric disorders in general are prevalent in the bariatric population [6], including higher rates of a history of sexual abuse, as well as higher rates of posttraumatic stress disorder (PTSD), which are also more prevalent in the veteran population [9, 10]. Active substance abuse is readily monitored and screened for using routine toxicology tests that provide quantitative, binary results. Thus, it is possible to insist on preoperative abstinence from illicit drug or alcohol use, and compliance can be closely followed before and after surgery.

Our results support the results of other bariatric surgery programs treating a nonveteran population. Heinberg and Ashton examined excess weight loss in patients who have a history of drug and/or alcohol abuse compared to those with no such history [11]. They showed that early weight loss is equivalent for those with and without a history of substance abuse. However, at 6, 9, and 12 months, the substance abuse groups lost significantly more weight than the control group. The present study showed greater weight loss in the SA group compared to the NA group (RYGB, LSG only), but this difference was not significant, possibly due to the small sample size. In addition, the rates of prior substance abuse in the Heinberg and Ashton study were much lower than in this study (10.9% versus 36%, resp.), perhaps reflecting the difference between the general and the veteran populations. In another study of 80 bariatric patients followed for two years, Clark et al. reported a history of drug or alcohol abuse in 13% [12]. They also found that the history of abuse translated favorably into greater weight loss after two years. One potential reason SA patients do just as well postoperatively as the NA counterparts may be due to past treatment received for their substance abuse disorders. Most substance abuse interventions involve learned coping skills and utilizing social support as an alternative to relying on addictive behaviors for managing stress. These skills may be useful in the postoperative bariatric population as well. In addition, the SA patients are required to have a period of sobriety prior to surgery. As a result, they have demonstrated the ability to make positive health behavior changes, which may be indicative of their ability to follow a strict postoperative weight management plan.

 The relatively small sample sizes, particularly in the laparoscopic gastric band group, increase the risk of type II error in this study. While we found no difference in percent EWL between the SA and NA groups, perhaps a larger sample size would demonstrate a difference. 

 Recent attention has been given to the “addictive behavior” of obese patients and the replacement of one addiction (i.e., food) for another (e.g., alcohol) [13, 14]. However, Suzuki et al. found the prevalence of alcohol use disorders among patients who have undergone bariatric surgery to be similar to the general population. Nonetheless, they found more postoperative alcohol use disorders in those patients who had a prior history of abuse, compared to those with no history of abuse [15]. This is consistent with our findings of a six-fold increased risk of recurrent abuse in patients with a prior preoperative history of drug or alcohol abuse. Others have found that weight loss surgery, in and of itself, predisposes the patients to substance abuse after bariatric surgery in the period 12 to 24 months postoperatively [16, 17]. Although it appears that the patients with a history of SA were more likely to develop substance abuse postoperatively in our study, the number of cases was too small to identify a statistically significant difference. In addition, the overall low rates of postoperative substance abuse may reflect the close involvement of behavioral medicine specialists in our clinic, throughout the postoperative course. A larger study with a more prolonged postoperative followup course will be needed to elucidate this point.

## 5. Conclusions

 A prior history of substance abuse is common in veterans undergoing bariatric surgery. Nonetheless, bariatric surgery results in significant weight loss that is comparable to the general veteran bariatric cohort. The rate of substance abuse is low postoperatively and not significantly different compared to patients without prior substance abuse, over a follow-up period of up to nine years. Close and prolonged multidisciplinary followup throughout the postoperative course is likely to prove being integral to success for patients with a prior history of substance abuse.

## Figures and Tables

**Table 1:** Demographic data comparing cohorts.

	SA = 74	NA = 131	*P*
Age (years)	51	52	0.672
Male/female	86.5/13.5%	73.3/26.7%	0.018
Preoperative BMI (kg/m^2^)	46.2	46.2	0.901
LGB	11 (14.9%)	12 (9.2%)	0.097
LSG	22 (29.8%)	50 (38.2%)	0.081
RYGB	41 (55.3%)	69 (52.6%)	0.095

SA: cohort with prior history of substance abuse.

NA: cohort with no prior history of substance abuse.

BMI: body mass index.

LGB: laparoscopic gastric banding.

LSG: laparoscopic sleeve gastrectomy.

RYGB: Roux-en-Y gastric bypass.

NS: not significant.

**Table:** (a)

LGB	SA	NA	*P*
%EWL at 6 months	25.4 ± 20.7%	26.4 ± 12.5%	0.88
%EWL at 12 months	33.4 ± 25.2%	34.0 ± 27.2	0.96

**Table:** (b)

LSG	SA	NA	*P*
%EWL at 6 months	55.0 ± 25.7%	46.7 ± 17.5%	0.18
%EWL at 12 months	59.6 ± 25.2	57.3 ± 21.8%	0.75

**Table:** (c)

RYGB	SA	NA	*P*
%EWL at 6 months	62.5 ± 19.4%	60.6 ± 23.6%	0.72
%EWL at 12 months	75.8 ± 23.5%	69.5 ± 19.9%	0.15

SA: cohort with prior history of substance abuse.

NA: cohort with no prior history of substance abuse.

LGB: laparoscopic gastric banding.

LSG: laparoscopic sleeve gastrectomy.

RYGB: Roux-en-Y gastric bypass.

%EWL: percent excess weight loss.

**Table 3:** Substance abuse recidivism after bariatric surgery in patients with and without a prior history of substance abuse.

	Total	SA group	NA group	*P*
	205	74 (36.1%)	131 (63.9%)	
Postoperative substance abuse	8	6 (8.1%)	2 (1.5%)	0.234

SA: cohort with prior history of substance abuse.

NA: cohort with no prior history of substance abuse.

NS: not significant.
